# Unintended effects of proton pump inhibitors (PPIs) in patients with glioblastoma (GBM): A double-edged sword

**DOI:** 10.1093/nop/npac035

**Published:** 2022-05-27

**Authors:** Michael P Castro

**Affiliations:** Beverly Hills Cancer Center, Beverly Hills, California, USA; Personalized Cancer Medicine, PLLC Los Angeles, California, USA; Cellworks Group, Inc. S. San Francisco, California, USA

A vast literature demonstrates that aldehyde dehydrogenase-1A1 (ALDH1A1) mediates therapy resistance and is associated with poor prognosis across human cancers. In glioblastoma (GBM), ALDH1A1 expression above vs below the mean causes temozolomide and radiation resistance and is associated with a substantial reduction in survival (methylated MGMT 14.6 vs 32.9 months, *P* = .004; unmethylated MGMT: 12.6 vs 21.4 months *P* = .005), while knockdown of ALDH1A1 restores sensitivity to chemotherapy and radiation therapy.^1,2^ ALDH1A1 is also a known mediator of resistance to EGFR (epidermal growth factor receptor) blockade and an activator of HIF1A. Aldehyde dehydrogenases detoxify anticancer drugs and function as antioxidants by reversing lipid peroxidation and repairing etheno-DNA adducts, thus maintaining REDOX homeostasis that confers chemotherapy and radiation failure.^3^ ALDH1A1 also catalyzes the conversion of retinaldehyde to retinoic acid resulting in the stemness phenotype that causes tumor re-population.^4^ These functions of ALDH1A1 make it a target of interest in two ongoing clinical trials of disulfiram in newly diagnosed and relapsed GBM.

Against this background, recent observations that proton pump inhibitors (PPIs) are potent inducers of ALDH1A1 activity indicates a potential source of iatrogenesis. Omeprazole binds to the aldehyde binding site of ALDH1A1 thereby upregulating enzyme activity by 4- to 6-fold, highlighting its utility to lower oxidative stress.^5^ Pantoprazole is also reported to enhance ALDH1A1 gene expression by 3.73- to 3.85-fold.^6^ Lansoprazole upregulates NRF2 and leads to a reduction of oxidative stress,^7^ representing another antioxidant mechanism that subverts cytotoxicity. Other effects of PPIs on the tumor microenvironment (TME), gut microbiome, and YAP oncogene activation suggest that they are not strictly benign agents.

By contrast, PPI use in oncology is buttressed by laboratory observations that PPIs may have anti-tumor benefits. Hypothetically, blocking vacuolar-ATPase proton pumps (ATP6V0A1/2) deactivate the pH inversion that acidifies the TME and raises intracellular pH. Evidence suggests these effects diminish invasion in GBM cell lines,^8^ promote apoptosis, and enhance chemotherapy uptake (Table 1). Clinical trials in breast cancer and head and neck cancer have found some benefits for PPIs, although their safety and utility for patients with GBM have not been prospectively assessed.

**Table 1.** Competing Consequences of PPI Use on Tumor Biology

Effect	Pathways
PRO	PPIs → ATPV0A1/2↓ → pH inversion↓ → MMP2/9↓→ [invasion, migration]↓
	PPIs → ATPV0A1/2↓ → pH inversion↓ → [chemotherapy uptake]↑
CON	PPIs → ALDH1A1↑ → ROS↓ → [ferroptosis↓, DNA repair↑] → radiation/chemotherapy resistance
	PPIs → ALDH1A1↑ → [retinaldehyde > retinoic acid (RA)]↑ → RXR/RAR → [stemness, metastases, immune editing]

Abbreviations: ALDH1A1, aldehyde dehydrogenase-1; MMP, matrix metalloproteinases; PPIs, proton pump inhibitors; ROS, reactive oxygen species; RXR/RAR, retinoid X receptor/retinoid acid receptor.

Does the impact of PPIs on ALDH1A1 imply a need for immediate change in the clinical reflex of routinely prescribing PPIs for neuro-oncology patients? At the moment, no randomized data are available to definitively address the question for patients with glioma. Potentially, electronic medical records data could be correlated with survival endpoints to explore the question. In the meantime, clinicians are left only with the literature related to ALDH1A1 in GBM biology and therapy resistance to decide if the apparent risks are worth the utility of these agents in managing the risks of upper gastrointestinal (GI) bleeding and hyperacidity. While the knowledge base is incomplete, the potential for causing treatment failure and worsening survival appears substantial.

As alternative therapy, H2 blockers have been shown to block tumor invasion and increase survival in in vivo laboratory models of GBM,^9^ also by blocking pH inversion and tumor invasiveness. Aldehyde dehydrogenases are upregulated by histamine and downregulated by H1R and H2R antagonists, including cimetidine.^10^ Therefore, H2 blockers could be considered for GI prophylaxis in place of PPIs.

The recent lessons about PPIs and ALDH1A1 counter enthusiasm for using PPIs in oncology. Their complex biology calls for caution with regard to repurposing drugs for anticancer benefits. Pleotropic drug effects arising from promiscuous protein binding can foster both pro- and anti-tumoral effects. The observations about ALDH1A1 activation by at least some PPIs present an opportunity to remove potentially harmful drugs that diminish treatment efficacy and survival for patients with GBM. Based on a deep understanding of ALDH1A1 in cancer biology, such an opportunity should not be lost while we search for confirmatory patient data to definitively inform clinical practice. Until more is known, the possibility that PPIs produce unintended harm should prompt caution and a change in their reflexive use in neuro-oncology.
